# Anticancer Alkaloid Lamellarins Inhibit Protein Kinases

**DOI:** 10.3390/md20080026

**Published:** 2008-10-07

**Authors:** Dianne Baunbæk, Nolwenn Trinkler, Yoan Ferandin, Olivier Lozach, Poonsakdi Ploypradith, Somsak Rucirawat, Fumito Ishibashi, Masatomo Iwao, Laurent Meijer

**Affiliations:** 1 C.N.R.S., Cell Cycle Group, Station Biologique, B.P. 74, 29682 Roscoff Cedex, Bretagne, France; 2 Laboratory of Medicinal Chemistry, Chulaborhn Research Institute, Vipavadee-Rangsit Highway, Bangkok 10210, Thailand; 3 Division of Marine Life Science and Biochemistry, Faculty of Fisheries, Nagasaki University, 1-14 Bunkyo-machi, Nagasaki 852-8521, Japan; 4 Department of Applied Chemistry, Faculty of Engineering, Nagasaki University, 1-14 Bunkyo-machi, Nagasaki 852-8521, Japan

**Keywords:** lamellarin, kinase inhibitor, cyclin-dependent kinases, CK1, DYRK-1A, GSK-3

## Abstract

Lamellarins, a family of hexacyclic pyrrole alkaloids originally isolated from marine invertebrates, display promising anti-tumor activity. They induce apoptotic cell death through multi-target mechanisms, including inhibition of topoisomerase I, interaction with DNA and direct effects on mitochondria. We here report that lamellarins inhibit several protein kinases relevant to cancer such as cyclin-dependent kinases, dual-specificity tyrosine phosphorylation activated kinase 1A, casein kinase 1, glycogen synthase kinase-3 and PIM-1. A good correlation is observed between the effects of lamellarins on protein kinases and their action on cell death, suggesting that inhibition of specific kinases may contribute to the cytotoxicity of lamellarins. Structure/activity relationship suggests several paths for the optimization of lamellarins as kinase inhibitors.

## 1. Introduction

Marine organisms constitute an original and relatively untapped source of new enzymes and novel drugs of great biotechnological and pharmaceutical applications potential [reviewed in 1–2]. Cephalosporins, ara-C and ara-A represent the first molecules of marine origin which have reached the market. Quite a few molecules derived from marine organisms are now being investigated in clinical tests, essentially against cancer, inflammation, chronic pain and neurodegenerative diseases. In the cancer field, ecteinascidin 743 (yondelis), dehydrodidemnin B (aplidine), bryostatin-1, dolastatin and analogs, ziconotide (Prialt), discodermolide are some of the most advanced compounds. Other molecules, rather than being investigated as potential clinical drugs, have been developed as important pharmacological tools for cell biology, such as okadaic acid, kainic acid, latrunculin, tetrodotoxin, saxitoxin.

Among the promising anti-cancer drug candidates derived from marine invertebrates is the family of lamellarins [reviewed in 3–5]. These hexacyclic pyrrole alkaloids were first isolated in 1985 from a prosobranch mollusk of the genus *Lamellaria* [6]. They were later extracted from and identified in various species of ascidians [7–14] and sponges [15–16] collected from very diverse areas. Over 38 lamellarins (A–Z and α–γ) have been described [reviewed in 4, 5]. Following the discovery of the potent anti-proliferative and pro-apoptotic activities of lamellarins [17–24], their biological activities have been extensively studied. Lamellarins are potent inhibitors of topoisomerase I [19–21], they interact with DNA [19] and they target mitochondria directly and induce the release of cytochrome C and apoptosis-inducing factor (AIF) [23, 24]. They also function as multi-drug resistance reversal drugs [17, 22]. Furthermore, Lamellarin α 20-sulfate inhibits HIV-1 integrase [14, 26, 27].

In the course of screening for pharmacological inhibitors of disease-relevant protein kinases such as cyclin-dependent kinases (CDKs) [28, 29], glycogen synthase kinase-3 (GSK-3) [30], PIM1 [31], “dual-specificity, tyrosine phosphorylation regulated kinase 1A” (DYRK1A) [32–34], casein kinase 1 (CK1) [35], we discovered that several lamellarins inhibit the catalytic activity of some of these kinases. We here report on the kinase inhibitory activity of 22 lamellarins [18, 36, 37] on 6 protein kinases. These lamellarins were also tested in parallel for their effects on the survival of human neuroblastoma SH-SY5Y cells and the expression of a selection of key proteins. The contribution of kinase inhibition to the anti-tumor properties of lamellarins is discussed.

CDK1/cyclin B is essential for G1/S and G2/M phase transition of the cell cycle. Inhibition of CDK1/cyclin B leads to cell cycle arrest eventually leading ultimately to cell death. Deregulation of CDK5/p25 has been associated with neurodegenerative diseases including Alzheimer’s disease, therefore it was included in the panel of kinases tested. In addition to inactivating glycogen synthase, GSK-3α/ß is also implicated in control of the cellular response to DNA damage and is directly involved in Alzheimer’s disease. PIM-1 is up-regulated in prostate cancers. DYRK1A, suspected to play a role in Down’s syndrome and Alzheimer’s disease, is thought to participate in central nervous system development, in growth control, and development. Likewise, CK1 is implicated in regulation of various physiological processes, and in diseases such as cancers and Alzheimer’s disease.

## 2. Results and Discussion

### 2.1. Lamellarins inhibit protein kinases

While screening marine natural products for new chemical inhibitors of protein kinases, we found lamellarin D to display significant activity. We thus initially assembled a small collection of natural and synthetic lamellarin analogs (Table 1).

All compounds were run on our kinase assay panel comprising CDK1/cyclin B, CDK5/p25, GSK-3α/ß, PIM-1, DYRK1A, CK1. Results are presented in Table 2. They show that some lamellarins are potent inhibitors of various protein kinases. The limited number of lamellarin analogs precludes a solid structure/activity relationship study. Nevertheless, striking differences in kinase inhibitory activity are observed following minor changes at the lamellarin chemical structure. For example,

removal of the hydroxyl at R1 modestly modifies the activity (except GSK-3, DYRK1A and CK1), compare lamellarin D (**1**) and lamellarin 4 (**10**).
removal of the hydroxyl at R2 results in minor changes in activity (except DYRK1A and CK1), compare lamellarin D (**1**) and lamellarin 3 (**9**).
removal of the hydroxyl at R3 results in major reduction in activity, compare lamellarin D (**1**) and lamellarin 7 (**13**)
removal of the hydroxyl at R4 results in enhanced inhibitory activity, compare lamellarin D (**1**) and lamellarin 6 (**12**).
O-methylation at R6 results in massive loss of inhibitory activity, compare lamellarin D (**1**) and lamellarin α (**2**).
replacing the hydroxyl groups of lamellarin D (**1**) at R_1,_ R_3,_ and R_6_ by O-isopropyl completely abolishes inhibitory activity, see lamellarin 31 (**18)** and 33 (**19**). This is also the case when substituting hydroxyl to isopropyl at positions R_1,_ R_4,_ and R_6_ of lamellarin N (**6**), lamellarin 32 (**21**) and 34 (**20**).
transposition of the substitutions at R_3_ and R_4_ of lamellarin D (**1**), resulting in lamellarin N (**6**), lead to 10-fold enhanced kinase inhibitory activity.
Reduction in activity due to saturation of D-ring double bond (C5=C6) has previously been reported to be due to loss of planarity and therefore steric hindrance in ATP pocket of targets [19]. Kinase inhibitory activities of lamellarins are generally reduced when the D-ring double bond is saturated, compare lamellarins D (**1**) with di-H-lamellarin (**3**), and lamellarins N (**6**) with lamellarin L (**7**).

Altogether these results suggest complex but specific interactions between lamellarins’ susbstituents and their kinase targets. Co-crystal structure would be most helpful to understand these interactions and optimize lamellarins as kinase inhibitors.

### 2.2. Selectivity of lamellarins

Although most active compounds were active on all six kinases, a few lamellarins displayed apparent selectivity towards some kinases, suggesting that some degree of selectivity might be gained following the synthesis of more analogs. We next tested the selectivity of lamellarin N on the Cerep kinase selectivity panel (Table 3). Results show that lamellarin N does not inhibit all, but displays some selectivity for a few kinases, some of which are major cancer targets (VEGFR1/2, Flt-3, PDGFR, Lck, Lyn). Inhibition of protein kinases by the tested compounds observed could also possibly be due to non-specific interactions, raising the need for more analogues having a higher specificity for a limited amount of kinases. Hopefully more selective lamellarin analogues can be designed when their interaction mode will be better understood.

### 2.3. Cell death induction by lamellarins

We next tested the effects of each lamellarin, at an initial 10 μM concentration, on the survival of the neuroblastoma SH-SY5Y cell line after 48 h exposure (Table 2). Cell survival was estimated by the MTS reduction assay. Several compounds showed clear effects on the SH-SY5Y cell survival rate. A complete dose-response curve was performed for these active compounds after 24 h and 48 h exposure and the IC_50_ values were calculated (Table 2). The most active compounds were lamellarin D (**1**) (IC_50_: 0.019 μM), lamellarin N (**6**) (IC_50_: 0.025 μM), lamellarin 3 (**9**) (IC_50_: 0.056 μM), and lamellarin 6 (**12**) (IC_50_: 0.11 μM). These lamellarins share a hydroxyl group at R1 and R6, an O-methyl group at R5, and a double bond between C5 and C6. As observed with the kinase inhibitory activity, a saturated C5-C6 bond instead of a double bond leads to an decrease in activity (compare compounds **1** and **3**, **4** and **5**, **6** and **7**). Lamellarins which were totally inactive on kinases were devoid of effects on cell death. Altogether these first results suggest that kinase inhibition may contribute to the effects of lamellarins on cell proliferation and cell death.

**Figure 1.**
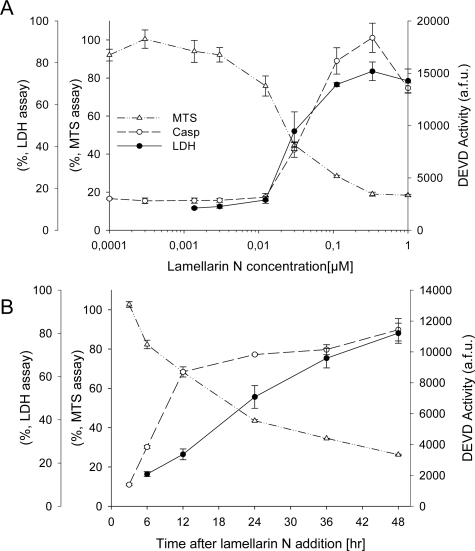
**Dose- and time- dependent induction of cell death by lamellarin N.** (A) Neuroblastoma SH-SY5Y cells were treated with lamellarin N at various concentrations. Cell death was measured by LDH release, cell survival was assessed by the MTS reduction assay, and caspase 3 activity was monitored as DEVDase activity. MTS and LDH assay were carried out 48 hours after treatment with lamellarin N. The caspase assay was carried out 24 hours after treatment. (B) Kinetics of cell survival, cell death and caspases activation following exposure of SH-SY5Y cells to 1 μM lamellarin N. Average of 3 independent experiments performed in triplicate. The standard deviation (±SD) is indicated by error bars.

SH-SY5Y cells were next incubated with a range of lamellarin N concentrations or at 1 μM over 48 hrs. Cell survival was monitored by the MTS reduction assay, cell death was assessed by the LDH release assay and caspase activation was measured using DEVD as a substrate (Figure 1). Kinetics and dose-response curves show that cells start dying rapidly after lamellarin N (half-life: 20 hours) at doses as low as 0.05 μM.

**Figure 2.**
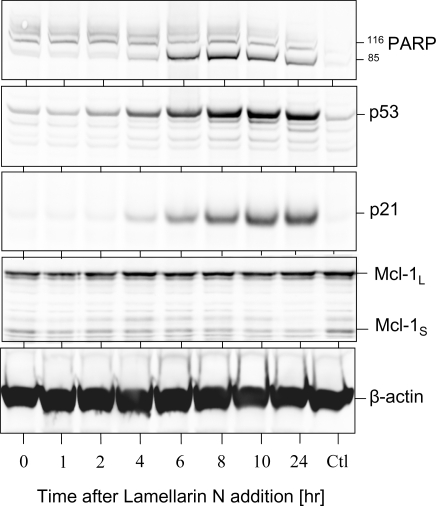
**Lamellarin N triggers PARP cleavage, p53 & p21^CIP1^ upregulation, but not Mcl-1 down-regulation.** SH-SY5Y cells treated at time 0 with 1 μM lamellarin N. Cells were harvested at different time-points and protein extracted for SDS-PAGE followed by Western blot analysis using antibodies directed against PARP, p53, p21^CIP1^, or Mcl-1, as described in the materials and methods section. β-actin was used as loading control. “Ctrl” denoted untreated sample after 10 hours.

Expression of p53, p21^CIP1^ & Mcl-1 and PARP cleavage were next evaluated by Western blotting (Figure 2). Induction of PARP cleavage and p53 and p21 expression already takes place after 6 hour treatment with lamellarin N (Figure 2) and at concentration as low as 0.1 μM (data not shown). Interestingly, in contrast to the effects observed with roscovitine and other CDK inhibitors [42], the survival factor Mcl-1 is not down-regulated (Figure 2). Thus lamellarin N is able to trigger cell death in the presence of a constant level of the survival factor Mcl-1. As Mcl-1 confers resistance to the BCL-2 selective antagonist ABT-737 and to the proteasome inhibitor bortezomib [43], lamellarins might favorably combine with these treatments.

## 3. Conclusions

In conclusion, we have identified new molecular targets of lamellarins. Combined with the well-supported effects of lamellarins on topoisomerase 1, kinase inhibition may underlie the promising anti-tumor properties of lamellarins. This work is now currently being extended in several directions: (i) synthesis and biological evaluation of new analogs, to allow the identification of more potent and more selective lamellarins as kinase inhibitors; (ii) optimization on the most cancer-relevant kinases; (ii) identification of the molecular mechanism of action of lamellarins on kinases (enzymological analysis, co-crystal structures); (iv) investigation of the contribution of kinase inhibition to the promising anti-tumoral effects of lamellarins (and the synergy with previously identified mechanisms of action); (v) conversely, identification of lamellarin analogues devoid of kinase inhibitory properties but still able to interact with topoisomerase 2, possibly leading to compounds with less toxic side effects. Identification of the key kinase targets of biologically active lamellarins or, in contrast, elimination of the kinase inhibition properties of lamellarins should be used to optimize this family of compounds towards selective and potent anti-tumor agents.

## 5. Experimental Procedures

### 5.1. Chemistry

Compounds **2**, **4**, **5** (lamellarin N), **6**, **7** and **17**–**20** were synthesized employing Hinsberg-type pyrrole formation and Suzuki-Miyaura cross-couplings as the key reactions as previously described [37]. Compounds **1**, **3** and **8**–**16** were synthesized by the previous methods in which the pyrrole core was constructed by N-ylide mediated intramolecular condensation [18, 36].

### 5.2. Kinase preparation and assays

Kinase activities were assayed in buffer A (10 mM MgCl_2_, 1 mM EGTA, 1 mM DTT, 25 mM Tris-HCl pH 7.5, 50 μg heparin/ml) or C (60 mM β-glycerophosphate, 15 mM *p*-nitrophenyl phosphate, 25 mM MOPS (pH 7.2), 5 mM EGTA, 15 mM MgCl_2_, 1 mM dithiothreitol, 1 mM sodium vanadate, 1 mM phenylphosphate, 0.1 % Nonidet P-40), at 30°C, at a final ATP concentration of 15 μM. Blank values were subtracted and activities calculated as pmoles of phosphate incorporated during a 30 min. incubation. The activities were expressed in % of the maximal activity, *i.e.* in the absence of inhibitors. Controls were performed with appropriate dilutions of dimethylsulfoxide. Unless otherwise stated, the P81 phosphocellulose assay was used.

*CDK1/cyclin B* was extracted in homogenization buffer (60 mM ß-glycerophosphate, 15 mM p-nitrophenylphosphate, 25 mM Mops (pH 7.2), 15 mM EGTA, 15 mM MgCl_2_, 1 mM DTT, 1 mM sodium vanadate, 1 mM NaF, 1 mM phenylphosphate, 10 μg leupeptin/ml, 10 μg aprotinin/ml, 10 μg soybean trypsin inhibitor/ml and 100 μM benzamidine) from M phase starfish (*Marthasterias glacialis*) oocytes and purified by affinity chromatography on p9^CKShs1^-sepharose beads, from which it was eluted by free p9^CKShs1^ as previously described [38]. The kinase activity was assayed in buffer C, with 1 mg histone H1 /ml, in the presence of 15 μM [γ-^33^P] ATP (3,000 Ci/mmol; 1 mCi/ml) in a final volume of 30 μl. After 30 min. incubation at 30°C, 25 μl aliquots of supernatant were spotted onto 2.5 × 3 cm pieces of Whatman P81 phosphocellulose paper, and, 20 sec. later, the filters were washed five times (for at least 5 min. each time) in a solution of 10 ml phosphoric acid/liter of water. The wet filters were counted in the presence of 1 ml ACS (Amersham) scintillation fluid.

*CDK5/p25* was reconstituted by mixing equal amounts of recombinant human CDK5 and p25 expressed in *E. coli* as GST (Glutathione-S-transferase) fusion proteins and purified by affinity chromatography on glutathione-agarose (vectors kindly provided by Dr. L.H. Tsai) (p25 is a truncated version of p35, the 35 kDa CDK5 activator). Its activity was assayed with histone H1 in buffer C as described for CDK1/cyclin B.

*GSK-3 α/β* was purified from porcine brain by affinity chromatography on immobilized axin [39]. It was assayed, following a 1/100 dilution in 1 mg BSA/ml 10 mM DTT, with 5 μl 4 μM GS-1, a GSK-3 selective substrate, (YRRAAVPPSPSLSRHSSPHQSpEDEEE, obtained from Millegen (31682 Labège, France), in buffer A, in the presence of 15 μM [γ-^33^P] ATP in a final volume of 30 μl. After 30 min. incubation at 30°C, 25 μl aliquots of supernatant were processed as described above.

*PIM1* was expressed as a GST-fusion protein in *E. coli* and purified by affinity chromatography on glutathione-agarose. Its kinase activity was assayed for 30 min. with histone H1 in buffer C as described for CDK1/cyclin B.

*DYRK1A* was expressed as a GST fusion protein in *E. coli* (vector kindly provided by Dr. W. Becker, Institute for Pharmacology and Toxicology, Aachen, Germany) and affinity purified on glutathione-agarose. Its kinase activity was assayed in buffer C, with 0.16 mg myelin basic protein (MBP)/ml, in the presence of 15 μM [γ-^33^P] ATP in a final volume of 30 μl. After 30 min incubation at 30°C, 25 μl aliquots of supernatant were treated as described above.

*CK1* was purified from porcine brain by affinity chromatography on immobilized axin fragment [40]. The mixture of native CK1 isoforms (essentially CK1δ and CK1ɛ) was assayed in three-fold diluted buffer C, using 25 μM CKS peptide, a CK1 selective substrate (RRKHAAIGpSAYSITA, synthesized by Millegen (Labège, France). Assays were performed for 30 min. at 30°C, as described for GSK-3.

### 5.3. Cell viability and cell death

SH-SY5Y human neuroblastoma cells were grown in DMEM supplemented with 2mM L-glutamine (Invitrogen, Cergy Pontoise, France), plus antibiotics (penicillin-streptomycin) and a 10% volume of fetal calf serum (FCS) (Invitrogen). Cell viability was determined by means of the MTS (3- (4,5-dimethylthiazol-2-yl)-5- (3-carboxymethoxyphenyl)-2- (4-sulfophenyl)-2*H* -tetrazolium) reduction method as previously described [41]. Cell death was (also) assayed by LDH release. Lactate dehydrogenase is a stable cytosolic enzyme that is released upon cell lysis [40]. SH-SY5Y cells were also subjected to caspase activity assays. These are based on cleavage of a synthetic caspase substrate DEVD (MP Biomedicals, 199423), a subtrate of active caspase 3/6/7. The assay was carried out according to manufacturer’s instructions [41].

### 5.4. Protein extraction, SDS-PAGE and Western Blotting

At given time points and lamellarin doses, SH-SY5Y cells were harvested in phosphate buffered saline (PBS) with commercially available protease inhibitors (Roche, 11836145001). Cells were homogenized by sonication in homogenization buffer supplemented with 0.1% NP-40 and protease inhibitors. The homogenization buffer consist of: 25mM MOPS, 15mM EGTA, 15mM MgCl_2_, 1mM sodium orthovanadate, 1mM sodium fluoride, 60mM β-glycerophosphate, 2mM DTT, 15mM p-nitrophenylphosphate, and 1mM phenyl phosphate-di-sodium salt (all reagents supplied by Sigma). Sonication retrieved proteins. Cellular proteins were subjected to electrophoresis in SDS 10% polyacrylamide gels and transferred to a PVDF membrane (Invitrogen). Membranes were incubated with primary antibodies directed against p53 (Santa Cruz Biotech, sc-263) (1:1000), p21^CIP1^ (Calbiochem, OP64) (1:1000) or poly (ADP-ribose) polymerase (PARP) (Santa Cruz Biotech, sc-7150) (1:500). Appropriate secondary antibodies conjugated to horseradish peroxidase (Biorad) were added to visualize the proteins using the ECL detection system.

## Figures and Tables

**Table 1.** **Structure of the lamellarins used in this study**. A single (——) or a double (=) bond is present between C5 and C6, depending on the molecule. Me, methyl; i-Pr, isopropyl. **22**: -OH at position 7. 
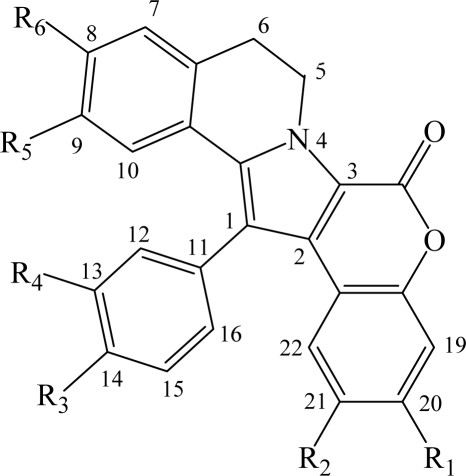

#	Lamellarin	R1	R2	R3	R4	R5	R6	5–6
**1**	lamellarin D	OH	OMe	OH	OMe	OMe	OH	=
**2**	lamellarin α	OH	OMe	OH	OMe	OMe	OMe	=
**3**	di-H-lamellarin D	OH	OMe	OH	OMe	OMe	OH	=
**4**	lamellarin H	OH	OH	OH	OH	OH	OH	=
**5**	di-H-lamellarin H	OH	OH	OH	OH	OH	OH	=
**6**	lamellarin N	OH	OMe	OMe	OH	OMe	OH	=
**7**	lamellarin L	OH	OMe	OMe	OH	OMe	OH	=
**8**	lamellarin G tri-OMe	OMe	OMe	OMe	OMe	OMe	OMe	=
**9**	lamellarin 3	OH	H	OH	OMe	OMe	OH	=
**10**	lamellarin 4	H	OMe	OH	OMe	OMe	OH	=
**11**	lamellarin 5	OH	OMe	OMe	OMe	OMe	OMe	=
**12**	lamellarin 6	OH	OMe	OH	H	OMe	OH	=
**13**	lamellarin 7	OH	OMe	H	OMe	OMe	OH	=
**14**	lamellarin 8	H	H	OH	OMe	OMe	OH	=
**15**	lamellarin 9	H	H	OH	OH	OH	OH	=
**16**	lamellarin 11	H	H	OMe	OMe	OMe	OMe	=
**17**	lamellarin 12	O-CH_2_-O		OMe	OMe	OMe	OMe	=
**18**	lamellarin 33	O*i*-Pr	OMe	O*i*- Pr	OMe	OMe	O*i*-Pr	=
**19**	lamellarin 31	O*i*-Pr	OMe	O*i*-Pr	OMe	OMe	O*i*-Pr	=
**20**	lamellarin 34	O*i*-Pr	OMe	OMe	O*i*-Pr	OMe	O*i*-Pr	=
**21**	lamellarin 32	O*i*-Pr	OMe	OMe	O*i*-Pr	OMe	O*i*-Pr	=
**22**	lamellarin K	OH	OMe	OH	OMe	OMe	OMe	=

**Table 2.** **Biological activity of lamellarins.**Each lamellarin was tested on 6 protein kinases. Enzyme activities were assayed as described in the Experimental section. Results are reported as IC_50_ values (expressed in μM) estimated from the dose-response curves. -, no inhibitory activity was detected (highest concentration tested is indicated in parentheses). Lamellarins were also tested for their effect on the survival of human neuroblastoma SH-SY5Y cells, using the MTS assay (IC_50_ values expressed in μM). The effect of some lamellarins on HeLa cells [18] is provided for comparison. Nt, not tested.

#	Lamellarin	CDK1/cyclin B	CDK5/p25	GSK-3α/ß	PIM 1	DYRK1A	CK1	SH-SY5Y	HeLa [18]
**1**	lamellarin D	0.50	0.55	0.3	0.10	0.45	13.0	0.019	0.011
**2**	lamellarin α	8.0	> 10	1.4	0.59	5.0	7.9	− (10)	
**3**	di-H-lamellarin D	1.85	0.11	0.9	0.20	0.50	5.9	0.41	nt
**4**	lamellarin H	− (10)	− (10)	9.5	− (10)	− (10)	5.3	0.45	> 100
**5**	di-H-lamellarin H	− (10)	− (10)	0.67	− (10)	− (10)	5.2	2.55	nt
**6**	lamellarin N	0.070	0.025	0.005	0.055	0.035	− (10)	0.025	nt
**7**	lamellarin L	0.38	0.1	0.041	0.25	0.14	− (10)	0.7	nt
**8**	lamellarin G tri-								
	OMe	− (10)	− (10)	− (10)	− (10)	> 10	− (10)	− (100)	nt
**9**	lamellarin 3	0.53	0.60	0.58	0.15	0.06	0.41	0.056	0.04
**10**	lamellarin 4	2.0	0.6	0.05	0.05	0.08	1.3	0.79	0.85
**11**	lamellarin 5	− (10)	− (10)	− (10)	2.0	− (10)	− (10)	8.0	2.5
**12**	lamellarin 6	0.10	0.03	0.13	0.33	0.09	0.8	0.11	0.04
**13**	lamellarin 7	4.3	2.1	2.1	− (10)	− (10)	− (10)	0.14	0.07
**14**	lamellarin 8	5	0.9	2.2	0.7	1.0	− (10)	2.65	4.0
**15**	lamellarin 9	− (10)	− (10)	− (10)	− (10)	− (10)	− (10)	− (10)	1.1
**16**	lamellarin 11	− (10)	− (10)	− (10)	− (10)	− (10)	− (10)	− (10)	5.7
**17**	lamellarin 12	− (10)	− (10)	− (10)	− (10)	− (10)	− (10)	− (10)	> 100
**18**	lamellarin 33	− (10)	− (10)	− (10)	− (10)	− (10)	− (10)	− (100)	nt
**19**	lamellarin 31	− (10)	− (10)	− (10)	− (10)	− (10)	− (10)	− (100)	nt
**20**	lamellarin 34	− (10)	− (10)	− (10)	− (10)	− (10)	− (10)	− (100)	nt
**21**	lamellarin 32	− (10)	− (10)	− (10)	− (10)	− (10)	− (10)	− (100)	nt
**22**	lamellarin K	− (10)	− (10)	− (10)	0.6	− (10)	6.0	− (30)	nt

**Table 3.** **Kinase inhibition selectivity of lamellarin N evaluated on the CEREP Kinase Selectivity Panel (44 kinases).**Preparation and assay of kinases are described [44] (www.cerep.com). Enzymes were assayed in the presence of 10 μM lamellarin N, and kinase activities expressed as % of control kinase activity, *i.e.* in the absence of inhibitor. ≥ 80 % inhibition at 10 μM is underlined in grey.

Protein Kinase	Activity (% of control)	SEM (%)
Abl kinase (h)	61.4	0.8
Akt1/PKB α (h)	104.6	2.6
AMPKα	42.4	0.9
BMX kinase (h) (Etk)	60.8	5.4
Brk (h)	72.8	4.7
CaMK2α (h)	38.2	2.2
CaMK4 (h)	89.9	3.1
CDC2/CDK1 (h) (cycB)	20.4	0.6
CDK2 (h) (cycE)	57.1	0.3
CHK1 (h)	93.3	1.9
CHK2 (h)	47.6	0.6
c-Met kinase (h)	92.1	1.6
CSK (h)	47.4	2.5
EphB4 kinase (h)	89.7	0.9
ERK1 (h)	94.1	0.1
ERK2 (h) (P42mapk)	79.9	1.4
FGFR2 kinase (h)	19.8	0.6
FGFR4 kinase (h)	62.5	0.3
FLT-1 kinase (h) (VEGFR1)	4.0	0.2
FLT-3 kinase (h)	0.4	0.4
Fyn kinase (h)	20.1	1.2
IGF1R kinase (h)	87.2	1.6
IRK (h) (InsR)	46.7	1.6
JNK 2 (h)	15.7	1.8
KDR kinase (h) (VEGFR2)	7.4	1.1
Lck kinase (h)	7.1	0.7
Lyn kinase (h)	7.2	0.5
MAPKAPK2 (h)	96.6	2.1
MEK1/MAP2K1 (h)	81.5	1.0
p38α kinase (h)	97.4	1.4
p38δ kinase (h)	96.0	0.9
p38γ kinase (h)	79.6	0.6
PDGFRβ kinase (h)	1.8	0.4
PDK1 (h)	89.8	0.6
PKA (h)	80.0	4.6
PKCα (h)	91.7	7.6
PKCβ 1 (h)	102.0	1.8
PKCγ (h)	94.1	1.9
Ret kinase (h)	1.8	0.4
ROCK2 (h)	103.3	0.9
RSK2 (h)	44.5	0.5
Src kinase (h)	56.2	0.4
Syk (h)	97.1	0.7
TRKA (h)	0.8	0.3

## Abbreviations:

CDK: cyclin-dependent kinase
CK1: casein kinase-1
DYRK1A: dual-specificity tyrosine-phosphorylated and regulated kinase 1A
FCS: fetal calf serum
GSK-3: glycogen synthase kinase-3
GST: Glutathione-S-transferase
MBP: myelin basic protein
MTS: 3- (4,5-dimethylthiazol-2-yl)-5- (3-carboxymethoxyphenyl)-2- (4-sulfophenyl)-*2H*–tetrazolium
PARP: poly (ADP-ribose) polymerase

## References

[1] Bourguet-Kondracki ML, Kornprobst JM (2005). Marine pharmacology: potentialities in the treatment of infectious diseases, osteoporosis and Alzheimer's disease. Adv. Biochem. Eng. Biotechnol.

[2] Simmons TL, Andrianasolo E, McPhail K, Flatt P, Gerwick WH (2005). Marine natural products as anticancer drugs. Mol. Cancer Ther.

[3] Cironi P, Albericio F, Alvarez M (2004). Lamellarins: isolation, activity and synthesis. Progr. Heterocycl. Chem.

[4] Bailly C (2004). Lamellarins, from A to Z: a family of anticancer marine pyrrole alkaloids. Curr. Med. Chem. Anti-Cancer Agents.

[5] Fan RH, Peng J, Hamann MT, Hu JF (2008). Lamellarins and related pyrrole-derived alakaloids from marine organisms. Chem. Rev.

[6] Andersen RJ, Faulkner DJ, He CH, Van Duyne GD, Clardy J (1985). Metabolites of the marine prosobranch mollusk *Lamellaria* sp. J. Am. Chem. Soc.

[7] Lindquist N, Fenical W, Van Duyne GD, Clardy J (1988). New alkaloids of the lamellarin class from the marine ascidian *Didemnum chartaceum* (Sluiter, 1909). J. Org. Chem.

[8] Carroll AR, Bowden BF, Coll JC (1993). Studies of Australian ascidians. I. Six new lamellarin-class alkaloids from a colonial ascidian, *Didemnum* sp. Austr. J. Chem.

[9] Urban S, Capon RJ, Lamellarin S (1996). A New Aromatic Metabolite from an Australian Tunicate *Didemnum* sp. Aust. J. Chem.

[10] Reddy MVR, Faulkner DJ, Venkateswarlu Y, Rao MR (1997). New lamellarin alkaloids from an unidentified ascidian from the Arabian Sea. Tetrahedron.

[11] Davis RA, Carroll AR, Pierens GK, Quinn RJ (1999). New lamellarin alkaloids from the australian ascidian, *Didemnum chartaceum*. J. Nat. Prod.

[12] Ham J, Kang H (2002). A novel cytotoxic alkaloid of lamellarin class from a marine ascidian Didemnum sp. Bull. Korean Chem. Soc.

[13] Reddy SM, Srinivasulu M, Satyanarayana N, Kondapi AK, Venkateswarlu Y (2005). New potent cytotoxic lamellarin alkaloids from Indian ascidian *Didemnum obscurum.*. Tetrahedron.

[14] Reddy MV, Rao MR, Rhodes D, Hansen MS, Rubins K, Bushman FD, Venkateswarlu Y, Faulkner DJ (1999). Lamellarin alpha 20-sulfate, an inhibitor of HIV-1 integrase active against HIV-1 virus in cell culture. J. Med. Chem.

[15] Urban S, Butler MS, Capon RJ (1994). Lamellarins O and P: New Aromatic Metabolites from the Australian Marine Sponge *Dendrilla cactos*. Aust. J. Chem.

[16] Urban S, Hobbs L, Hooper JNA, Capon RJ (1995). Lamellarins Q and R: New Aromatic Metabolites from an Australian Marine Sponge *Dendrilla cactos*. Aus t. J. Chem.

[17] Quesada AR, Garcia Gravalos MDJL (1996). Fernandez Puentes Polyaromatic alkaloids from marine invertebrates as cytotoxic compounds and inhibitors of multidrug resistance caused by P-glycoprotein. Br. J. Cancer.

[18] Ishibashi F, Tanabe S, Oda T, Iwao M (2002). Synthesis and structure-activity relationship study of lamellarin derivatives. J. Nat. Prod.

[19] Facompre M, Tardy C, Bal-Mahieu C, Colson P, Perez C, Manzanares I, Cuevas C, Bailly C (2003). Lamellarin D: a novel potent inhibitor of topoisomerase I. Cancer Res.

[20] Tardy C, Facompre M, Laine W, Baldeyrou B, Garcia-Gravalos D, Francesch A, Mateo C, Pastor A, Jimenez JA, Manzanares I, Cuevas C, Bailly C (2004). Topoisomerase I-mediated DNA cleavage as a guide to the development of antitumor agents derived from the marine alkaloid lamellarin D: trimethylester derivatives incorporating amino acid residues. Bioorg. Med. Chem.

[21] Marco E, Laine W, Tardy C, Lansiaux A, Iwao M, Ishibashi F, Bailly C, Gago F (2005). Molecular determinants of topoisomerase I poisoning by lamellarins: comparison with camptothecin and structure-activity relationships. J. Med. Chem.

[22] Vanhuyse M, Kluza J, Tardy C, Otero G, Cuevas C, Bailly C, Lansiaux A (2005). Lamellarin D: a novel pro-apoptotic agent from marine origin insensitive to P-glycoprotein-mediated drug efflux. Cancer Lett.

[23] Kluza J, Gallego MA, Loyens A, Beauvillain JC, Sousa-Faro JM, Cuevas C, Marchetti P, Bailly C (2006). Cancer cell mitochondria are direct proapoptotic targets for the marine antitumor drug lamellarin D. Cancer Res.

[24] Gallego MA, Ballot C, Kluza J, Hajji N, Martoriati A, Castéra L, Cuevas C, Formstecher P, Joseph B, Kroemer G, Bailly C, Marchetti P (2008). Overcoming chemoresistance of non-small cell lung carcinoma through restoration of an AIF-dependent apoptotic pathway. Oncogene.

[25] Banwell MG, Hamel E, Hockless DC, Verdier-Pinard P, Willis AC, Wong DJ (2006). 4,5-Diaryl-1H-pyrrole-2-carboxylates as combretastatin A-4/lamellarin T hybrids: Synthesis and evaluation as anti-mitotic and cytotoxic agents. Bioorg. Med. Chem.

[26] Ridley CP, Reddy MV, Rocha G, Bushman FD, Faulkner DJ (2002). Total synthesis and evaluation of lamellarin alpha 20-Sulfate analogues. Bioorg. Med. Chem.

[27] Yamaguchi T, Fukuda T, Ishibashi F, Iwao M The first total synthesis of lamellarin α 20-sulfate, a selective inhibitor of HIV-1 integrase. Tetrahedron.

[28] Malumbres M, Barbacid M (2005). Mammalian cyclin-dependent kinases. Trends Biochem. Sci.

[29] Knockaert M, Greengard P, Meijer L (2002). Pharmacological inhibitors of cyclin-dependent kinases. Trends Pharmacol. Sci.

[30] Meijer L, Flajolet M, Greengard P (2004). Pharmacological inhibitors of glycogen synthase kinase-3. Trends Pharmacol. Sci.

[31] Bachmann M, Moroy T (2005). The serine/threonine kinase Pim-1. Int. J. Biochem. Cell Biol.

[32] Galceran J, de Graaf K, Tejedor FJ, Becker W (2003). The MNB/DYRK1A protein kinase: genetic and biochemical properties. J. Neural. Transm. Suppl.

[33] Hammerle B, Elizalde C, Galceran J, Becker W, Tejedor FJ (2003). The MNB/DYRK1A protein kinase: neurobiological functions and Down syndrome implications. J. Neural Transm. Suppl.

[34] Ferrer M Barrachina, Puig B, Martinez de Lagran M, Marti E, Avila J, Dierssen M (2005). Constitutive Dyrk1A is abnormally expressed in Alzheimer disease, Down syndrome, Pick disease, and related transgenic models. Neurobiol. Dis.

[35] Knippschild U, Wolff S, Giamas G, Brockschmidt C, Wittau M, Wurl PU, Eismann T, Stoter M (2005). The role of the casein kinase 1 (CK1) family in different signaling pathways linked to cancer development. Onkologie.

[36] Ishibashi F, Miyazaki Y, Iwao M (1997). Total syntheses of lamellarin D and H. The first synthesis of lamellarin-class marine alkaloids. Tetrahedron.

[37] Fujikawa N, Ohta T, Yamaguchi T, Fukuda T, Ishibashi F, Iwao M (2006). Total synthesis of lamellarin D, L, and N. Tetrahedron.

[38] Meijer L, Borgne A, Mulner O, Chong JPJ, Blow JJ, Inagaki N, Inagaki M, Delcros JG, Moulinoux JP (1997). Biochemical and cellular effects of roscovitine, a potent and selective inhibitor of the cyclin-dependent kinases cdc2, cdk2 and cdk5. Eur. J. Biochem.

[39] Primot, Baratte B, Gompel M, Borgne A, Liabeuf S, Romette JL, Costantini F, Meijer L (2000). Purification of GSK-3 by affinity chromatography on immobilised axin. Protein Expr. & Purif.

[40] Reinhardt J, Ferandin Y, Meijer L (2007). Purification CK1 by affinity chromatography on immobilised axin. Protein Expr. & Purif.

[41] Ribas J, Boix J (2004). Cell differentiation, caspase inhibition, and macromolecular synthesis blockage, but not BCL-2 or BCL-XL proteins, protect SH-SY5Y cells from apoptosis triggered by two CDK inhibitory drugs. Exp. Cell Res.

[42] Bettayeb K, Oumata N, Echalier A, Ferandin Y, Endicott J, Galons H, Meijer L (2008). CR8, a potent and selective, roscovitine-derived inhibitor of cyclin-dependent kinases. Oncogene.

[43] Nguyen M, Marcellus RC, Roulston A, Watson M, Serfass L, Murthy Madiraju SR, Goulet D, Viallet J, Bélec L, Billot X, Acoca S, Purisima E, Wiegmans A, Cluse L, Johnstone RW, Beauparlant P, Shore GC (2007). Small molecule obatoclax (GX15-070) antagonizes MCL-1 and overcomes MCL-1-mediated resistance to apoptosis. Proc. Natl. Acad. Sci. U.S.A.

[44] Bach S, Knockaert M, Lozach O, Reinhardt J, Baratte B, Schmitt S, Coburn SP, Tang L, Jiang T, Liang DC, Galons H, Dierick JF, Totzke F, Schächtele C, Lerman AS, Carnero A, Wan Y, Gray N, Meijer L (2005). Roscovitine targets: protein kinases and pyridoxal kinase. J. Biol. Chem.

